# Doxorubicin-Induced Translocation of mtDNA into the Nuclear Genome of Human Lymphocytes Detected Using a Molecular-Cytogenetic Approach

**DOI:** 10.3390/ijms21207690

**Published:** 2020-10-17

**Authors:** Tigran Harutyunyan, Ahmed Al-Rikabi, Anzhela Sargsyan, Galina Hovhannisyan, Rouben Aroutiounian, Thomas Liehr

**Affiliations:** 1 Department of Genetics and Cytology, Yerevan State University, Alex Manoogian 1, Yerevan 0025, Armenia; tigranharutyunyan@ysu.am (T.H.); angela.sargsyan@ysu.am (A.S.); galinahovhannisyan@ysu.am (G.H.); genetik@ysu.am (R.A.); 2 Institute of Human Genetics, Jena University Hospital, Friedrich Schiller University, Am Klinikum 1, D-07747 Jena, Germany; ahmedgenetic@hotmail.com

**Keywords:** CBMN assay, doxorubicin, FISH, mtDNA insertion in nuclei, micronuclei

## Abstract

Translocation of mtDNA in the nuclear genome is an ongoing process that contributes to the development of pathological conditions in humans. However, the causal factors of this biological phenomenon in human cells are poorly studied. Here we analyzed mtDNA insertions in the nuclear genome of human lymphocytes after in vitro treatment with doxorubicin (DOX) using a fluorescence in situ hybridization (FISH) technique. The number of mtDNA insertions positively correlated with the number of DOX-induced micronuclei, suggesting that DOX-induced chromosome breaks contribute to insertion events. Analysis of the odds ratios (OR) revealed that DOX at concentrations of 0.025 and 0.035 µg/mL significantly increases the rate of mtDNA insertions (OR: 3.53 (95% CI: 1.42–8.76, *p* < 0.05) and 3.02 (95% CI: 1.19–7.62, *p* < 0.05), respectively). Analysis of the distribution of mtDNA insertions in the genome revealed that DOX-induced mtDNA insertions are more frequent in larger chromosomes, which are more prone to the damaging action of DOX. Overall, our data suggest that DOX-induced chromosome damage can be a causal factor for insertions of mtDNA in the nuclear genome of human lymphocytes. It can be assumed that the impact of a large number of external and internal mutagenic factors contributes significantly to the origin and amount of mtDNA in nuclear genomes.

## 1. Introduction

The endosymbiotic hypothesis suggests that mitochondria were free-living cells that colonize at least one single cell. Millions of years of co-evolution resulted in the transfer of a large number of genes from mitochondria to the nucleus [1]. There are two types of mitochondrial DNA (mtDNA) in the nucleus. First are nuclear DNA sequences encoding proteins (~2000) for the proper function of mitochondria, as they are no longer present in the DNA of mitochondria. The second are nuclear DNA sequences that are copies of existing mtDNA. These sequences, known as nuclear DNA sequences of mitochondrial origin or NUMTs (pronounced “new-mights”), can be detected in at least 85 sequenced eukaryotic genomes [2]. Currently over 750 NUMTs have been identified in human reference genome and new insertions may occur at a rate of ~5 × 10^−6^ per germ cell per generation [1,3]. 

NUMTs have received considerable attention given that their de novo occurrence in the nuclear genome may contribute to the development of pathological conditions. Willett-Brozick et al. [4] described a 41 bp mtDNA insertion at the breakpoint junction of a familial constitutional reciprocal translocation between chromosomes 9 and 11. The implication of mtDNA insertion into the nuclear genome in the development of Pallister-Hall syndrome [5], severe plasma factor VII deficiency [6], and mucolipidosis-IV [7] was demonstrated in further studies. Frequent translocation of mtDNA into the nuclear genome in human cancer cells was demonstrated recently. Analysis of 587 pairs of cancer and normal whole-genome sequencing data revealed 10 primary cancers (1.8%, 10/559) and two cancer cell lines (7.1%, 2/28) with somatic mtDNA integrations into their nuclear genomes. The length of the transferred mtDNA fragments ranged from 148 bp to entire mitochondrial genomes (16.5 kb) [8]. Srinivasainagendra et al. [9] demonstrated that colorectal adenocarcinoma genomes, on average, contain up to 4.2-fold more somatic NUMTs than matched normal genomes. At the same time, colorectal tumors obtained from women contained more NUMTs than men (4.52-fold vs. 3.51-fold, respectively). Thus, the transfer process of mtDNA into the nuclear genome, known as numtogenesis, is involved in the development of cancer [10].

It has been suggested that the distribution of NUMTs is non-random and NUMTs tend to be localized in damage-prone regions of the nuclear genome, such as open chromatin and fragile sites [11,12]. In studies with yeast, it has been demonstrated that incorporation of fragmented mtDNA into the nuclear genome is mediated by non-homologous end joining (NHEJ), microhomology-mediated end-joining or homologous recombination during the repair of I-SceI nuclease-induced DNA double-stranded breaks (DSB) of nuclear DNA [13,14].

Thus, we hypothesized that agents capable of inducing DSBs (e.g., chemical and physical mutagens) can contribute to the migration of mtDNA into the nuclear genome in human cells. To test our hypothesis, the well-known antitumor drug doxorubicin (DOX) [15,16] was chosen as an inducer of DNA DSBs. The ability of doxorubicin to induce DSBs directly due to intercalation into DNA and inhibition of topoisomerase II, and indirectly by the generation of reactive oxygen species has been shown previously [17]. The successful application of fluorescence in situ hybridization (FISH) for examining the variation in mtDNA insertions in nuclear chromosomes of human [8], maize [18], and rat [19] was the reason for selecting this method in our study.

Thus, in this study, we analyzed the frequency of mtDNA translocation in the nuclear genome of DOX-treated and untreated cultured human whole blood cells using mtDNA FISH probe. The relationship between the level of DOX-induced chromosome damage and DOX-induced mtDNA insertions in chromosomes was analyzed.

## 2. Results

### 2.1. CBMN Assay in Human Whole Blood Lymphocytes 

As demonstrated previously, spontaneous insertions of mtDNA in human chromosomes is associated with chromosomal aberrations [8]. To develop the optimal conditions for the induction of chromosomal aberrations, the ability of different concentrations of DOX to induce micronuclei (MN) in human leukocytes was studied using cytokinesis-block micronucleus assay (CBMN). MN is a marker of chromosome damage that originates from chromosome fragments or whole chromosomes [20]. DOX has been shown to induce MN in human lymphocytes mainly through chromosome breakage and, to a lesser extent, through chromosome delay [20], and chromosome breaks are expected to facilitate mtDNA transfer in nuclear DNA. 

**Figure 1:**
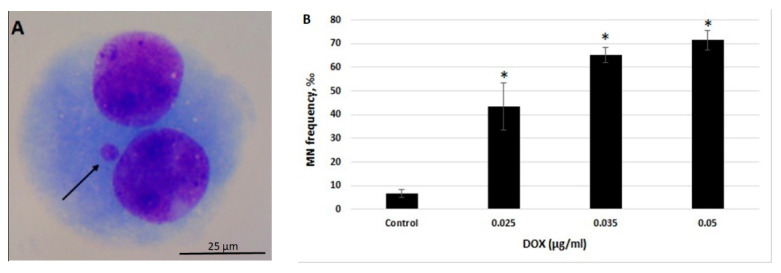
Genotoxic effect of doxorubicin (DOX) as analyzed by cytokinesis-block micronucleus (CBMN) assay in human peripheral blood lymphocytes. (**A**) Representative image of the binucleated cell with micronuclei (MN) (MN is indicated by the arrow; 100× oil objective, Giemsa staining). (**B**) Frequency of micronuclei in human peripheral blood lymphocytes treated with DOX at concentrations 0.025, 0.035, and 0.05 µg/mL. Statistically significant increase in MN levels is indicated at * *p* < 0.05. Scale bar = 25 μm.

Our preliminary studies demonstrated that treatment of human peripheral blood lymphocytes with DOX at concentrations of 0.07 µg/mL and higher, significantly suppress the frequency of dividing cells (data not shown). Thus, lower concentrations of DOX were used in this study (i.e., 0.025, 0.035 and 0.05 µg/mL). The obtained results indicate that DOX significantly elevates the number of MN in all of the studied concentrations (*p* < 0.05) (Figure 1A,B).

### 2.2. FISH Analysis of mtDNA Translocation in Metaphase Chromosomes

**Figure 2:**
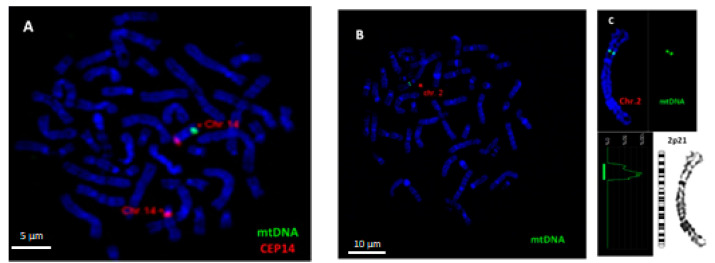
Fluorescence in situ hybridization (FISH) analysis of mtDNA insertion in chromosomes of human peripheral blood lymphocytes. (**A**) Hybridization of a homemade mtDNA probe (spectrum green) with chromosome 14q31 of a healthy person with confirmed mtDNA insertion (positive control). Localization of mtDNA insertion on chromosome 14 was confirmed using CEP 14 probe (spectrum orange). (**B**) mtDNA insertion (spectrum green) on chromosome 2 after treatment with DOX at a concentration of 0.025 µg/mL. (**C**) Karyotyping revealed the localization of mtDNA fragment on 2p21. Chromosomes are counterstained with DAPI. Scale bar = 5 μm,10 μm.

To visualize mtDNA insertions in metaphase chromosomes, FISH analysis was carried out using homemade probe for mtDNA. The specificity of FISH probe for mtDNA was confirmed using metaphase chromosomes of a healthy person with previously confirmed mtDNA insertion in chromosome 14q31, i.e., a cytogenetically visible NUMT (Figure 2A). An example of the insertion of mtDNA into chromosome 2p21 in DOX-treated cell is shown in Figure 2B,C.

**Figure 3:**
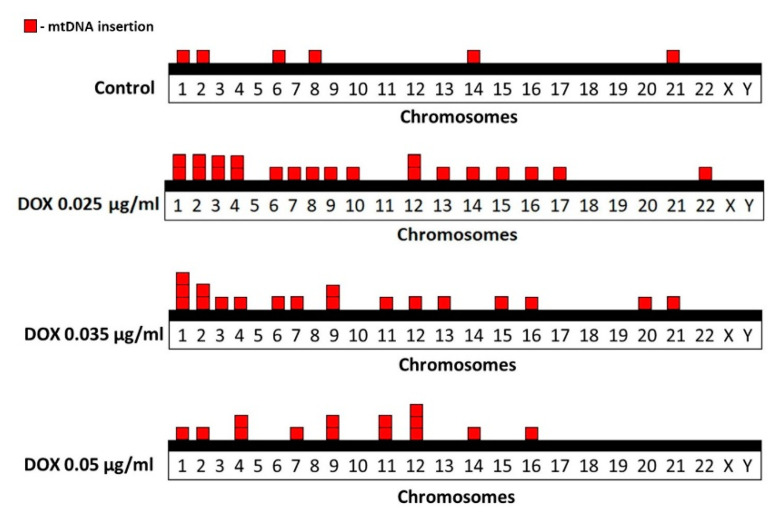
Distribution of spontaneous and DOX-induced mtDNA insertions in human metaphase chromosomes. Each square block corresponds to one insertion event.

FISH analysis of human metaphase chromosomes revealed genomic distribution of cytogenetically visible mtDNA insertions in control and DOX-treated cells (Figure 3). In the control group, six mtDNA insertions were identified on chromosomes 1, 2, 6, 8, 14 and 21. In cells treated with DOX at a concentration of 0.025 µg/mL in total 21 mtDNA insertions were identified on chromosomes 1, 2, 3, 4, 6, 7, 8, 9, 10, 12, 13, 14, 15, 16, 17 and 22. In cells treated with DOX at a concentration of 0.035 µg/mL in total 18 mtDNA insertions were identified on chromosomes 1, 2, 3, 6, 7, 9, 11, 12, 13, 15, 16, 20 and 21. In cells treated with DOX at a concentration of 0.05 µg/mL in total 14 mtDNA insertions were identified on chromosomes 1, 2, 4, 7, 9, 11, 12, 14 and 16.

FISH analysis of the control group revealed 0.0033 ± 0.0015 translocations of mtDNA into the nuclear genome per metaphase (Table 1). All DOX concentrations increased the level of cytogenetically visible mtDNA insertions; this effect was especially pronounced at concentrations of 0.025 and 0.035 µg/mL (*p* < 0.05) (Table 1). We also assessed the relationship between DOX treatment and mtDNA translocations in nuclei. The odds ratio of mtDNA translocations after treatment with DOX at concentrations of 0.025 and 0.035 µg/mL was 3.53 (95% CI: 1.42–8.76; *p* = 0.006) and 3.02 (95% CI: 1.19–7.62; *p* = 0.019), respectively, indicating that exposure to DOX significantly increases the probability of mtDNA insertions into nuclear genome (Table 1).

### 2.3. Correlation Studies of mtDNA Insertions

Since it was shown previously that the number of NUMTs in each chromosome correlates with chromosome size [21], we examined whether DOX-induced distribution of de novo mtDNA corresponds to the literature data. In addition, correlation analyses were performed to establish the relationship between DOX-induced mtDNA insertions and chromosome length and NUMTs per chromosome in reference human genome [21], gene density [22] and levels of DOX-induced MN obtained in our study (Table 2). 

The obtained results revealed a significant positive correlation between mtDNA insertion numbers induced by DOX at concentrations of 0.025 and 0.035 µg/mL and chromosome length (*r* = 0.631 and *r* = 0.502, respectively) and NUMTs per chromosome (*r* = 0.667 and *r* = 0.509, respectively). These results indicate the randomness of DOX-induced insertions of mtDNA in human chromosomes, which is consistent with the literature data [21]. A significant relationship was shown between mtDNA insertions and levels of MN, induced by DOX at concentrations of 0.025 and 0.05 µg/mL, indicating the potential influence of chromosome damage on insertion of mtDNA in the nuclear genome.

## 3. Discussion

Although de novo mtDNA transfer of mitochondrial DNA into the nuclear genome has been reported in cells of patients with various pathologies [5,6,7,8,10], the possibility of mutagen-induced nuclear transfer of mtDNA has not been comprehensively studied to our knowledge, with the only exception being transfer in chick embryos from eggs exposed to X-rays [23]. 

Despite multiple physical barriers that prevent nuclear and mitochondrial DNA interactions, there are several mechanisms that enable translocation of mtDNA or even whole mitochondria into the nucleus. However, the mechanisms and factors contributing to these events are relatively poorly understood. 

Here, for the first time, we analyzed DOX-induced mtDNA translocation in the nuclear genome in healthy human blood lymphocytes using FISH. The available data suggests that integration of mtDNA in the nuclear genome can occur when there is increased genomic instability in nuclear and mitochondrial genomes. DOX was chosen because of its ability to cause damage to both the nuclear [24,25] and mitochondrial [26,27] DNA.

We demonstrated that DOX is capable of inducing the insertion of mtDNA in human cells and the number of insertions has a significant relationship with the levels of DOX-induced MN. Since chromosome breaks prevail over lagging chromosomes in DOX-induced MN [20,28], our results indicate an association between mtDNA transfer and chromosome breaks or DNA DSBs. Our data is in accordance with previous studies indicating that mtDNA translocation in nuclear genome occurs after repair of DSBs in yeast cells. Ricchetti et al. [13] demonstrated that I-SceI endonuclease-induced DSBs activate non-homologous end joining in haploid *Saccharomyces cerevisiae* cells. Sequencing of re-ligated loci revealed single insertions of 47, 77 and 97 bp fragments of mitochondrial DNA indicating that DNA damage of nuclear genome is crucial for occurrence of NUMTs. Similar results were obtained using extrachromosomal DSB repair assay where fragments of mtDNA of *Schizosaccharomyces pombe* were captured and integrated in extrachromosomal construct during DNA damage repair [14]. Currently, alterations in the DNA strand integrity and genomic instability are considered to be the most important factors contributing to the integration of mtDNA fragments in nuclear genome both during evolution and in de novo formation, which is mediated by the NHEJ repair pathway [8,13,14,21]. Thus, we suggests that the NHEJ repair pathway of DOX-induced DSBs may have a significant impact on the integration of mtDNA in the nuclear genome.

A number of studies have indicated the role of tumor cell genomic instability for mtDNA transfer in nuclear genome. It has been shown that mtDNA translocation in nuclear genome may occur in colorectal cancer cells and is influenced by the pathological cancer stage [9]. An increase in the frequency of mtDNA insertions in the nuclear genome of tumors may occur due to the mitochondrial dysfunction and mitochondrial genome instability observed in cancer cells [29,30]. Along with a number of chromosomal aberrations, an overlapping sequence microhomology (from 1 to 4 bp) of 80% breakpoints was identified in human cancer cells with de novo NUMTs, indicating the important role of DNA sequence microhomology in mitochondrial-nuclear DNA integration events. The authors concluded that these features are characteristic of DSB repair by the NHEJ pathway [8]. Analysis of mtDNA insertions in nuclear genome of 2658 cancers across 38 tumor types (2.1% overall positive rate) revealed a variable rate of occurrence depending on the cancer tissue type [31]. In particular, mtDNA insertions were identified in HER2-positive breast cancers (16% cases) and lung cancers (14.6% cases). The integration sites of mtDNA fragments were spatially closer to inversion and translocation breakpoints in the studied cases. The authors suggested that the integration of mtDNA segments into nuclear DNA is often mechanistically combined with some specific processes underlying the structural variations in the nuclear genome [31]. 

We demonstrated that DOX-induced mtDNA insertions positively correlate with chromosome length. It is noteworthy that DOX-induced structural aberrations in human lymphocytes, of which 14% were chromatid aberrations and 2% were chromosome aberrations, mainly involve chromosomes 1, 2, 3, 4, 6 and 11 [32]. In our study, 45% of all mtDNA insertions involve these chromosomes. We also found a correlation between mtDNA insertions and NUMTs per chromosome in reference human genome. Therefore, our data confirm previous studies and demonstrate that larger chromosomes that harbor more NUMTs are more prone to incorporate new fragments of mtDNA [21]. 

It has been shown that in humans, NUMT integrations preferentially target coding or regulatory sequences of genes [33]. However, we did not find a correlation between gene density and DOX-induced mtDNA insertions in chromosomes. This is in accordance with Simone et al. [21] who demonstrated that spontaneous distribution of NUMTs is not biased by the presence of genes in chromosomes. Nevertheless, we used FISH analysis for detection of cytogenetically visible fragments of mtDNA insertions in the chromosomes, but this does not preclude the possibility of insertions of smaller fragments in the nuclear genome.

In addition to nuclear DNA, DOX was shown to damage mtDNA, which can also contribute to de novo NUMT events. Yin et al. [34] demonstrated that DOX is capable of inducing mitophagy and mtDNA damage in cardiomyocytes in vitro due to the accumulation of mitochondrial superoxide, decrease in mitochondrial membrane potential and increase in markers of authophagosome. The ability of DOX to damage mtDNA is also shown in vivo, which in turn was linked to cardiotoxic effects [26,27]. In yeast cells, an elevated rate of escape of mtDNA to the nucleus was observed due to mitophagy induced by mutations in *YME1* (yeast mitochondrial escape 1) gene [35]. A higher frequency of mtDNA transfer in nuclear genome was observed in human breast cancer cell line MCF-7 by inactivating *YME1L1* gene (a human homolog of yeast *YME1*) [9]. In addition, mutations of *YME1L1* gene in adult cardiomyocytes cause mitochondrial fragmentation, heart failure and premature death in mice [36]. Interestingly, in these cells a metabolic switch from fatty oxidation toward glucose utilization was observed, which is frequent in cancer cells (the Warburg effect). Therefore, mutations of *YME1L1* gene may also be related to certain metabolic features of cancer cells. Thus, we can assume that DOX-induced mtDNA translocation to the nucleus is potentially involved in DOX-induced cardiotoxicity. These events occur due to genotoxic insult in the nucleus and/or induction of mitophagy. Although for now it is only a hypothesis, further studies in this direction may reveal additional mechanisms of DOX-induced cardiotoxicity. 

## 4. Materials and Methods 

### 4.1. Human Whole Blood Cultures

Blood samples were collected by venipuncture from four healthy nonsmoking volunteers (two female and two male) aged 27–29 years, without any history of genotoxic exposure and pathological conditions prior to investigation, and with normal 46,XX and 46,XY karyotypes, respectively. This study was approved by the Ethics Committee of the National Center of Bioethics (Faculty of Biology, Yerevan State University, Yerevan, Armenia), and informed consent was obtained from all study donors. The venous blood (2 mL from each donor) was collected into vacutainers with heparin. A cell line with insertion of a cytogenetically visible NUMT in chromosome 14 was also used as positive control.

### 4.2. CBMN Assay

Cytokinesis-blocked micronucleus assay was performed for analysis of levels of DOX-induced chromosome damage in human whole blood lymphocytes according to Fenech [37]. Heparinized whole blood was added to RPMI 1640 (Gibco, Germany) medium (1:10) containing 10% fetal bovine serum (Biochrom, Cambridge, UK), 1% penicillin/streptomycin (Santa Cruz Biotechnology, Dallas, TX, USA), and 10 μg/mL phytohemagglutinin (Biochrom, Germany). 48 h after culture initiation DOX (Santa Cruz Biotechnology, Dallas, TX, USA) was added at concentrations of 0.025, 0.035 and 0.05 µg/mL and incubated for 24 h. The concentrations of DOX used in our study were based on previous reports on the clastogenic and aneugenic effects of DOX in human peripheral blood lymphocytes [20] and on our preliminary studies. Cytochalasin B (3 μg/mL; Santa Cruz Biotechnology) was added after 44 h of incubation in order to block cytokinesis and obtain binucleated cells. In total, blood cultures were incubated for 72 h at 37 °C. Hypotonic treatment was performed for 3 min in 0.075 M KCl (Merck, Germany) at 4 °C. Fixation was done twice using ice-cold (−20 °C) ethanol/acetic acid (3:1 v/v; Sigma Aldrich, Germany). Slides were prepared by prewashing with fixative and cell suspension was added from a very low distance. After air drying, slides were stained with 10% Giemsa (Santa Cruz Biotechnology, Dallas, TX, USA). To determine the MN frequency, at least 1000 binucleated cells were evaluated per each experimental point and per donor. 

### 4.3. Metaphase Chromosome Preparation

Metaphase chromosomes were prepared as previously described [38]. Forty-eight hours after culture initiation, DOX was added at concentrations of 0.025, 0.035 and 0.05 µg/mL and incubated for 24 h. Colcemid (0.1 μg/mL final concentration; Merck, Germany) was added to the culture 1.5 h before harvesting and incubated at 37 °C to achieve metaphase block. Cells were harvested at the end of cultivation and centrifuged at 1500×  rpm (7 min). The medium was completely removed except for about 0.5 mL of supernatant remaining above the cell pellet. Then, 10 mL of pre-warmed (37 °C) hypotonic solution (0.075 M KCl) was added to the tubes and the contents were mixed gently and incubated for 15 min at 37 °C. After centrifugation and discarding the supernatant, cells were fixed in 10 mL of ice-cold (−20 °C) fixative (methanol/glacial acetic acid, 3:1 *v*/*v*). After incubation for 10 min at room temperature, the cells were centrifuged, supernatant was discarded, and 10 mL of fixative was added. After the last centrifugation, cells were resuspended in a small amount of fixative and the suspension was dropped onto a microscope slide, which was prewashed with fixative. Then the slide was placed on a hotplate (51 °C) covered by wet tissue paper until the surface of the slide was dried.

### 4.4. FISH Probe Synthesis

FISH analysis was performed using homemade probes for mtDNA identification. L15900 (5′-TAAACTAATACACCAGTCTTGTAAACC-3′) and H00599 (5′-TTGAGGAGGTAAGCTACATAA-3′) pair of primers were used for amplification of mtDNA insert from pGEM vector. The total volume of PCR reaction was 50 µL, which contained 5 µL of Advantage 2 PCR Buffer (Takara, Kyoto, Japan), 4 µL of dNTP Mix, 5 pM/µL (2 µL) of each primer L15900/H00599, 1 µL of Advantage 2 Titanium Taq Polymerase Mix (Takara), 6 µL of template DNA (~50 pg) and 30 µL of H2O. PCR conditions for amplification of the mtDNA insert were as follows: denaturation at 95 °C for 2 min, followed by 32 cycles of 95 °C for 15 s, 56 °C for 30 s, 72 °C for 1 min 30 s, then 72 °C for 10 min and termination at 4 °C. Labeling was performed using nine pairs of primers specific for mtDNA amplification [39] with the PCR conditions mentioned above (Table 3). Each reaction mixture contained 2 µL of buffer, 2 µL of Label-mix (Atto488 NT Labeling Kit, Jena Bioscience, Jena, Germany), 2 µL of MgCl2 25 mM, 0.12 µL of AmpliTaq DNA Polymerase, 2 µL of fluorochromes and 12.08 µL of H_2_O. To establish the positive control, the obtained probes were hybridized with metaphase chromosomes of a healthy person with confirmed insertion of mtDNA on 14q31. 

### 4.5. FISH Analysis

Our approach was based on the previous application of FISH for detection of mtDNA insertion in interphase nuclei [8]. Slides were dehydrated with ethanol series (70, 95 and 100%) for 3 min and treated with pepsin at 37 °C in a water bath. After washing with PBS for 5 min at room temperature, slides were treated with post-fixation solution (5 mL paraformaldehyde + 4.5 mL PBS + 500 µL 1 M MgCl2) for 10 min at room temperature under a flow hood. Slides were washed in PBS for 5 min at room temperature following dehydration with ethanol series for 3 min. To the pre-warmed (81 °C) slides, 8 µL of the mtDNA or negative control probe was added in 10% dextran sulfate and covered with cover slips, sealed with rubber cement and incubated overnight at 37 °C in a humidified chamber. The cover slips were removed, and slides were washed with 0.4xSSC solution for 5 min at 65 °C in water bath. After the slides were washed with 4xSSC + Tween solution (100 mL 20xSSC + 400 mL + 250 µL Tween20) for 5 min on the shaker at room temperature, they were rinsed in PBS for 5 min and dehydrated with ethanol series for 3 min. Slides were dried in the dark following staining with 30 µL of DAPI. Slides were analyzed under Zeiss fluorescent microscope using 100× objective. Images were captured using a CCD camera and processed with ISIS MetaSystems (MetaSystems, Germany). At least 450 metaphases were scored in each variant.

### 4.6. Statistical Analysis

Statistical analysis of data in CBMN assay and comparisons of the mean numbers of mtDNA insertions in different treatment groups was performed using the Student’s *t*-test. Pearson’s correlation was applied for analysis of the relationship between number of mtDNA insertions after treatment with DOX and chromosome length, gene density, number of NUMTs per chromosome and MN. The odds ratio was calculated using MedCalc, a publicly available statistical software [40]. Statistical analysis was performed using the statistical package Statgraphics Centurion 16.2 (Statgraphics Technologies, USA) and a *p* value of <0.05 was considered statistically significant. 

## 5. Conclusions

In summary, we have shown that DOX at concentrations inducing chromosomal instability and detected by micronucleus test, is capable of promoting mtDNA translocation into nuclear genome of human lymphocytes, which is significantly correlated with DOX-induced micronuclei level. MtDNA insertions in human chromosomes may occur due to DOX-induced DNA breaks in mitochondrial and nuclear genomes which is followed by activation of the NHEJ repair pathway. Thus, for the first time, the impact of chemical mutagen on mtDNA insertion in human chromosomes was demonstrated. DOX-induced cytogenetically visible mtDNA insertions are more likely to occur in larger chromosomes, which are more prone to damage after DOX exposure. However, these findings do not preclude the possibility of the insertion of mtDNA into smaller chromosomes that are undetectable by FISH method. On the basis of our own and previous results it can be assumed that the translocation of mtDNA into nucleus could be involved in the cardiotoxic activity of DOX.

## Figures and Tables

**Table 1:** Cytogenetically visible mtDNA insertions in metaphase chromosomes of human leukocytes and their association with DOX treatment.

DOX Doses (µg/mL)	Frequency of mtDNA Insertion per Metaphase(Mean ± SD)	Odds Ratio (95% CI)
Control	0.0033 ± 0.0015	-
0.025	0.0116 ± 0.0023 ^a^	3.53 (1.42–8.76) ^b^
0.035	0.0100 ± 0.0015 ^a^	3.02 (1.19–7.62) ^b^
0.05	0.0078 ± 0.0031	2.34 (0.89–6.11)

^a^*p* < 0.05—significant difference compared to control. ^b^ *p* < 0.05—significant relationship between DOX treatment and mtDNA insertion.

**Table 2:** Pearson correlation coefficient (r) of DOX-induced mtDNA insertions in human chromosomes with chromosome length, gene density, NUMTs per chromosome and MN frequency.

	mtDNA Insertions			
	Control	DOX 0.025 µg/mL	DOX 0.035 µg/mL	DOX 0.05 µg/mL
Chromosome length	0.321	0.631 **	0.502 *	0.319
Gene density (gene/Mb)	−0.153	0.134	−0.06	−0.01
NUMTs per chromosome	0.361	0.667 **	0.509 *	0.376
Number of MN	0.886	0.985 *	0.592	0.956 *

Statistically significant correlations are indicated at * *p* < 0.05 and ** *p* < 0.01.

**Table 3:** Primer set for whole mtDNA genome amplification. The sequences of the forward and reverse primers, PCR product and primer lengths are presented [39].

Product Length (bp)	Sequence (5’–3’)	Primers pair Length (bp)
1822	for tagccatgcactactcaccaga rev ggatgaggcaggaatcaaagac	22
1758	for ctgtatccgacatctggttcct rev gtttagctcagagcggtcaagt	22
2543	for acttaagggtcgaaggtggatt rev tcgatgttgaagcctgagacta	22
3005	for aagtcaccctagccatcattcta rev gatatcatagctcagaccatacc	23
2709	for ctgctggcatcactatactacta rev gattggtgggtcattatgtgttg	23
1738	for cttaccacaaggcacacctaca rev ggcacaatattggctaagaggg	22
1866	for gtctggcctatgagtgactaca rev cagttcttgtgagctttctcgg	22
1853	for ctccctctacatatttaccacaac rev aagtcctaggaaagtgacagcga	24
1872	for gcaggaatacctttcctcacag rev gtgcaagaataggaggtggagt	22

## Abbreviations

CBMN CI DOX DSB FISH	Cytokinesis-blocked micronucleus Confidence interval Doxorubicin DNA double-stranded breaks Fluorescence in situ hybridization
mtDNA	Mitochondrial DNA
NHEJ NUMT PCR RPMI	Non-homologous end joining Nuclear DNA sequences of mitochondrial origin Polymerase chain reaction Roswell Park Memorial Institute
